# *Bacillus cereus* Isolated From Vegetables in China: Incidence, Genetic Diversity, Virulence Genes, and Antimicrobial Resistance

**DOI:** 10.3389/fmicb.2019.00948

**Published:** 2019-05-15

**Authors:** Pengfei Yu, Shubo Yu, Juan Wang, Hui Guo, Ying Zhang, Xiyu Liao, Junhui Zhang, Shi Wu, Qihui Gu, Liang Xue, Haiyan Zeng, Rui Pang, Tao Lei, Jumei Zhang, Qingping Wu, Yu Ding

**Affiliations:** ^1^Department of Food Science and Technology, Institute of Food Safety and Nutrition, Jinan University, Guangzhou, China; ^2^State Key Laboratory of Applied Microbiology Southern China, Guangdong Provincial Key Laboratory of Microbial Culture Collection and Application, Guangdong Open Laboratory of Applied Microbiology, Guangdong Institute of Microbiology, Guangzhou, China; ^3^College of Food Science, South China Agricultural University, Guangzhou, China

**Keywords:** *Bacillus cereus*, food-borne pathogen, vegetables, incidence, MLST

## Abstract

*Bacillus cereus* is a food-borne opportunistic pathogen that can induce diarrheal and emetic symptoms. It is widely distributed in different environments and can be found in various foods, including fresh vegetables. As their popularity grows worldwide, the risk of bacterial contamination in fresh vegetables should be fully evaluated, particularly in vegetables that are consumed raw or processed minimally, which are not commonly sterilized by enough heat treatment. Thereby, it is necessary to perform potential risk evaluation of *B. cereus* in vegetables. In this study, 294 *B. cereus* strains were isolated from vegetables in different cities in China to analyze incidence, genetic polymorphism, presence of virulence genes, and antimicrobial resistance. *B. cereus* was detected in 50% of all the samples, and 21/211 (9.95%) of all the samples had contamination levels of more than 1,100 MPN/g. Virulence gene detection revealed that 95 and 82% of the isolates harbored *nheABC* and *hblACD* gene clusters, respectively. Additionally, 87% of the isolates harbored *cytK* gene, and 3% of the isolates possessed *cesB*. Most strains were resistant to rifampicin and β-lactam antimicrobials but were sensitive to imipenem, gentamicin, ciprofloxacin, kanamycin, telithromycin, ciprofloxacin, and chloramphenicol. In addition, more than 95.6% of the isolates displayed resistance to three kinds of antibiotics. Based on multilocus sequence typing, all strains were classified into 210 different sequence types (STs), of which 145 isolates were assigned to 137 new STs. The most prevalent ST was ST770, but it included only eight isolates. Taken together, our research provides the first reference for the incidence and characteristics of *B. cereus* in vegetables collected throughout China, indicating a potential hazard of *B. cereus* when consuming vegetables without proper handling.

## Introduction

*Bacillus cereus* is a Gram-positive, spore-forming opportunistic pathogen that is widespread in different environments and known to cause foodborne outbreaks in humans (Bottone, 2010; Osimani et al., 2018). *B. cereus* in food products at concentrations exceeding 10^4^ spores or vegetative cells per gram can cause food poisoning (Ehling-Schulz et al., 2006; Fricker et al., 2007; Meldrum et al., 2009). Prevalence of potential emetic and diarrheal *B. cereus* in different foods has been reported in Finland (Shaheen et al., 2010), Belgium (Rajkovic et al., 2006), Thailand (Chitov et al., 2008), the United Kingdom (Altayar and Sutherland, 2006; Meldrum et al., 2009), the United States (Ankolekar et al., 2009), South Korea (Park et al., 2009), and Africa (Ouoba et al., 2008). *B. cereus* is also one of the most prevalent foodborne pathogens in France and China (Glasset et al., 2016; Paudyal et al., 2018). From 1994 to 2005, 1,082 food poisoning cases caused by foodborne pathogens had been reported in China. *B. cereus* caused 145 (13.4%) of these cases, leading to six deaths (Wang et al., 2007).

Vegetables are an indispensable part for human food and nutrition. The World Health Organization recommends taking 400 g of fresh vegetables and fruits daily to promote human health (World Health Organization, 2003). Vegetables are often consumed directly or only with minimal processing that does not eliminate pathogenic bacteria, such as *B. cereus* (Goodburn and Wallace, 2013; Mogren et al., 2018). Since consumption of fresh vegetable has increased dramatically over the last few decades (Olaimat and Holley, 2012; Hackl et al., 2013), more foodborne outbreaks resulting from contaminated vegetables have simultaneously emerged (Berger et al., 2010; Castro-Ibáñez et al., 2016). For example, food poisoning outbreaks associated with vegetables contaminated by foodborne pathogens in Korea increased from 119 in 1998 to 271 in 2010 (Park et al., 2018). Therefore, it is necessary to monitor the contamination level of *B. cereus* in vegetables.

Consuming food contaminated by *B. cereus* can lead to gastrointestinal diseases, including diarrhea and emesis. Diarrhea is caused by different enterotoxins, including non-hemolytic enterotoxin (Nhe; Ehling-Schulz et al., 2006), hemolysin BL (Hbl; Ehling-Schulz et al., 2006), and cytotoxin K (CytK; Fagerlund et al., 2004), and emesis is due to a thermo- and acidic-stable non-ribosomal peptide, cereulide, which is encoded by the *ces* gene cluster (Ehling-Schulz et al., 2005; Ehlingschulz et al., 2015). In addition, *B. cereus* can induce other non-gastrointestinal-tract infections (Bottone, 2010; Rishi et al., 2013) and may even lead to death (Lund et al., 2000; Posfay-Barbe et al., 2008).

Antimicrobial treatment is the main method to eliminate foodborne pathogens, including *B. cereus*, in patients with food poisoning. However, antibiotic resistance in *B. cereus* has already emerged due to the abuse of antibiotics. The therapeutic effect of some antibiotics against antimicrobial-resistant isolates decreases or even disappears, leading to the failure of clinical treatment (Brown et al., 2003; Friedman, 2015; Torkar and Bedenić, 2018). As the consumption of vegetables contaminated with antimicrobial-resistant isolates may lead to more severe infection (Berthold-Pluta et al., 2017), it is important to test the antibiotic resistance of *B. cereus* in vegetables for food safety and human health.

*B. cereus* is widely distributed in nature and can contaminate foods primarily through soil and air (European Food Safety Authority [EFSA], 2005; Arnesen et al., 2008). Vegetables are generally planted in fields, where they are exposed to soil, and they are exposed to air during transportation and sale; they can therefore be easily contaminated by this pathogenic bacterium. *B. cereus* contamination in vegetables has been reported. The contamination rate in different vegetables ranged from 29.0 to 70.0% in South Korea (Chon et al., 2012, 2015; Kim H. J. et al., 2016; Kim Y. J. et al., 2016). In Mexico City, *B. cereus* was identified in 57% of the 100 analyzed samples (Flores-Urban et al., 2014). The contamination rate in different vegetables in southeast of Spain varied greatly (Valero et al., 2002).

As an essential daily nutrient, to date, no study has evaluated the occurrence rate of *B. cereus* in vegetables accounting for the whole of China. Therefore, in this study, we analyzed the contamination, genotypic diversity, pathogenic potential, and antimicrobial resistance of *B. cereus* isolated from vegetables in China to obtain an overview on the potential risk.

## Materials and Methods

### Vegetable Sample Collection

A total of 419 vegetable samples (89 *Coriandrum sativum L* samples, 85 var. *ramosa Hort.* samples, 134 *Cucumis sativus L.* samples, and 111 *Lycopersicon esculentum Mill.* samples) were collected from the local markets and supermarkets of 39 major cities in China (Table 1 and Supplementary Figure S1) from 2011 to 2016, according to the general sample collection guidelines of the National Food Safety Standard (The Hygiene Ministry of China, 2010b). The samples were placed in sealed bags, transferred to the laboratory in a low-temperature (below 4°C) sampling box, and immediately subjected to microbiological analysis after sending back to the laboratory.

**Table 1:** Prevalence and contamination level of *B. cereus* in different vegetables.

Type of vegetable	Contamination rate (%)^a^	MPN value (MPN/g)^b^		
		MPN < 3 (%)	3 ≤ MPN < 1100 (%)	1100 ≤ MPN (%)
*Coriandrum sativum L*	56/89 (62.92)	1/56 (1.79)	38/56 (67.86)	17/56 (30.36)
*var. ramosa Hort.*	49/85 (57.65)	5/49 (10.20)	41/49 (83.67)	3/49 (6.12)
*Cucumis sativus L.*	63/134 (47.01)	12/63 (19.05)	50/63 (79.37)	1/63 (1.59)
*Lycopersicon esculentum Mill.*	43/111 (38.74)	19/43 (44.19)	24/43 (55.81)	0/43 (0.00)
Total	211/419 (50.36)	37/211 (17.54)	153/211 (72.51)	21/211 (9.95)

*^a^Contamination rate = number of positive samples/total samples. ^b^MPN value (MPN/g) = most probable number of B. cereus per gram sample.*

### Isolation and Identification of *B. cereus*

Twenty-five grams of vegetable samples was cut into pieces, transferred into a sterile homogenizer containing 225 ml phosphate buffered saline (PBS, 0.01 mol/L), and then homogenized at 8,000 rpm for 2 min using a rotary blade homogenizer. The homogenized solution and its 1/10 and 1/100 dilutions were used to detect *B. cereus* qualitatively and quantitatively according to the *B. cereus* test rules given by the National Food Safety Standard (The Hygiene Ministry of China, 2010a), as described previously (Gao et al., 2018). Mannitol yolk polymyxin (MYP) agar plate test, parasporal crystal observation (to distinguish between *B. cereus* and *Bacillus thuringiensis*), root growth observation, hemolysis test, catalase test, motility test, nitrate reduction test, casein decomposition test, lysozyme tolerance test, glucose utilization test, and acetyl methyl alcohol test were conducted for species detection. The most probable number (MPN) method was adopted for quantitative detection of species. Briefly, 1 ml of the homogenized solution and its 1/10 and 1/100 dilutions were inoculated into three tubes each containing 10 ml peptone soy polymyxin broth medium. The nine cultures were incubated at 30°C for 48 h. Then, the cultures were streaked onto MYP plates and incubated at 30°C for at least 24 h. Presumptive colonies were picked for species identification. MPN was determined based on the MPN table provided by the National Food Safety Standard (The Hygiene Ministry of China, 2010a) and the number of positive culture(s) to calculate the MPN of *B. cereus* per gram sample (MPN/g).

### Detection of Emetic and Enterotoxin Toxin Genes

Genomic DNA was obtained using the HiPure Bacterial DNA Kit (Magene, United States) following the manufacturer’s specifications. Polymerase chain reaction (PCR) amplification was conducted to detect the cereulide synthetase gene (*cesB*) and seven enterotoxin genes (*nheA, nheB, nheC, hblA, hblC, hblD*, and *cytK*) with a 20 μl reaction mixture consisting of 50 ng genomic DNA, 12.5 μl PCR Premix TaqTM (Takara, China), and 2 μM of each primer (Hansen and Hendriksen, 2001; Fagerlund et al., 2004; Ehling-Schulz et al., 2005; Oltuszak-Walczak and Walczak, 2013). The primers used in this study are listed in Table 2.

**Table 2:** Primers used in this study.

Primer	Sequence (5′–3′)	Target fragment length (bp)	Annealing temperature (°C)	References
HblA-F	GTGCAGATGTTGATGCCGAT	320	55	Hansen and Hendriksen, 2001
HblA-R	ATGCCACTGCGTGGACATAT			
HblC-F	AATGGTCATCGGAACTCTAT	750	55	Hansen and Hendriksen, 2001
HblC-R	CTCGCTGTTCTGCTGTTAAT			
HblD-F	AATCAAGAGCTGTCACGAAT	430	55	Hansen and Hendriksen, 2001
HblD-R	CACCAATTGACCATGCTAAT			
NheA-F	TACGCTAAGGAGGGGCA	500	55	Hansen and Hendriksen, 2001
NheA-R	GTTTTTATTGCTTCATCGGCT			
NheB-F	CTATCAGCACTTATGGCAG	770	55	Hansen and Hendriksen, 2001
NheB-R	ACTCCTAGCGGTGTTCC			
NheC-F	CGGTAGTGATTGCTGGG	583	55	Hansen and Hendriksen, 2001
NheC-R	CAGCATTCGTACTTGCCAA			
cytK-F	AAAATGTTTAGCATTATCCGCTGT	238	55	Oltuszak-Walczak and Walczak, 2013
cytK-R	ACCAGTTGTATTAATAACGGCAATC			
cesB-F	GGTGACACATTATCATATAAGGTG	1271	58	Ehling-Schulz et al., 2005
cesB-R	GTAAGCGAACCTGTCTGTAACAACA			
glpF-F	GCGTTTGTGCTGGTGTAAGT	549	59	PubMLST (http://pubmlst.org/bcereus/info/primers.shtml)
glpF-R	CTG CAATCGGAAGGAAGAAG			
gmk-F	TTAAGTGAGGAAGGGTAGG	600	56	
gmk-R	AATGTTCACCAACCACAA			
ilvD-F	GGGCAAACATTAAGAGAA	556	58	
ilvD-R	TTCTGGTCGTTTCCATTC			
pta-F	AGAGCGTTTAGCAAAAGAA	576	56	
pta-R	CAATGCGAGTTGCTTCTA			
pur-F	GCTGCGAAAAATCACAAA	536	56	
pur-R	CACGATTCGCTGCAATAA			
pycA-F	GTTAGGTGGAAACGAAAG	550	57	
pycA-R	CGTCCAAGTTTATGGAAT			
tpi-F	CCAGTAGCACTTAGCGAC	553	58	
tpi-R	GAAACCGTCAAGAATGAT			

### Antimicrobial Resistance Testing

The Kirby-Bauer disk diffusion method was employed to evaluate the antimicrobial resistant, intermediate, and sensitive profiles of the isolates to 20 selected antibiotics as previously described (The Clinical and Laboratory Standards Institute [CLSI], 2010; Gao et al., 2018). The zone diameter interpretive standards were referred to the standard for *Staphylococcus aureus* (The Clinical and Laboratory Standards Institute [CLSI], 2010).

### Multilocus Sequence Typing (MLST) Gene Amplification, Sequencing, and Determination

Seven housekeeping genes, namely *glp*, *gmk*, *ilvD*, *pta*, *pur*, *pycA*, and *tpi*, were amplified with different primers and conditions (Table 2) according to MLST protocol for *B. cereus* in PubMLST^1^. The sequence of each PCR product was sequenced and submitted to the PubMLST database to get the corresponding allele number. The multilocus sequence type (ST) of each isolate was obtained by ranking and submitting seven housekeeping gene allele numbers. New STs were assigned by the MLST website administrator. A minimum spanning tree was constructed with PHYLOViZ 2.0 software (Instituto de Microbiologia, Portugal) according to the relationships between MLST alleles (Ribeiro-Gonçalves et al., 2016) and to visualize the relatedness and genetic diversity of different isolates.

## Results

### Prevalence Analysis of *B. cereus* in Vegetables

*B. cereus* was detected in 211 of 419 (50%) vegetable samples (Table 1), and the contaminated samples were distributed in all 39 different cities in China from where the samples were collected. The contamination rate was higher than 60% in 15 cities presented, and only three cities had a contamination rate below 20% (Supplementary Figure S1).

The positive rates of *B. cereus* were 62.92% (56/89) for *C. sativum L*, 57.65% (49/85) for var. *ramosa Hort.*, 47.01% (63/134) for *C. sativus L.*, and 38.74% (43/111) for *L. esculentum Mill.*, respectively. Contamination levels of 9.95% (21/211) of all the samples exceeded 1,100 MPN/g. Among all positive samples, the contamination levels for the *C. sativum L* (30.36%; 17/56) and var. *ramosa Hort.* (6.12%; 3/49) samples exceeded 1,100 MPN/g, which was higher than the contamination levels for the *C. sativus L.* and *L. esculentum Mill.* samples.

### Distribution of Virulence Genes Among *B. cereus* Isolates

**FIGURE 1:**
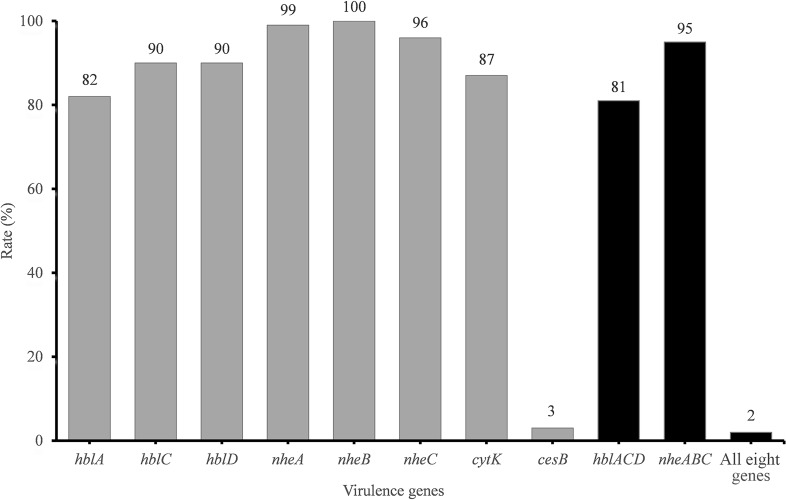
Detection rate of virulence genes in *B. cereus* from vegetables. The number at the top of the bars represents the positive rate of corresponding toxin genes. *hblACD* and *nheABC* mean that the strains are positive for *hblA*, *hblC*, and *hblD* or for *nheA*, *nheB*, and *nheC* at the same time, respectively. “All eight genes” presents the strains with all the detected toxin genes.

The presence of toxin genes is summarized in Figure 1. The cereulide synthetase gene *cesB* was detected in only 3% of isolates. In contrast, the rate of enterotoxin gene detection was very high. *hbl* genes encoding the Hbl toxin complex were detected in 81% of the samples. Additionally, 99, 100, and 96% of all isolates harbored *nheA*, *nheB*, and *nheC*, respectively. However, only 95% of the isolates harbored the integrated Nhe-encoding gene cluster *nheABC*. *cytK* was detected in 87% of the strains.

The virulence gene distribution could be divided into 20 different profiles. Only five isolates, namely, 2841-1B, 3713, 3715, 3715-2A, and 3740, possessed all eight virulence genes. Two isolates (3265 and 3463) harbored the least virulence gene list (*nheA-nheB*). The main gene profile (70.1% of all isolates) was *hblA*-*hblC*-*hblD*-*nheA*-*nheB*-*nheC*-*cytK*.

### Antimicrobial Susceptibility Test of *B. cereus* Isolates

**FIGURE 2:**
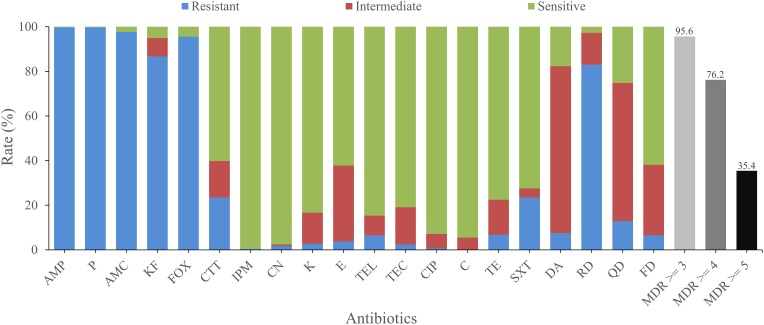
Antimicrobial resistance of *B. cereus* from vegetables. The blue, red, and green bars represent the proportion of resistant, moderately resistant, and sensitive strains, respectively. The light gray, gray, or dark bar represents the proportion of strains with multidrug resistance (MDR) to at least three, four, and five classes of antibiotics, respectively.

The antimicrobial susceptibilities of all isolates were tested with 20 antimicrobials. Most isolates were found to be resistant to amoxicillin-clavulanic (AMC; 97.6%), cephalothin (KF; 86.7%), penicillin (P; 99.7%), ampicillin (AMP; 99.7%), cefoxitin (FOX; 95.6%), which belong to β-lactams, as well as rifampin (RD, 83.0%), an ansamycin. On the other hand, most isolates were sensitive to some other antimicrobials, such as kanamycin (K; 83.3%), gentamicin (CN; 97.6%), telithromycin (TEL; 84.7%), imipenem (IPM; 99.7%), ciprofloxacin (CIP; 92.9%), chloramphenicol (C; 94.6%), and teicoplanin (TEC; 81.0%). Besides, most isolates exhibited intermediate resistance to quinupristin (QD; 61.9%) and clindamycin (DA; 74.8%; Figure 2).

There were 74 antimicrobial resistant profiles for all isolates. The strain 41-1 and 1515-1A turned out to be the most highly resistant isolates, which were resistant to 12 antibiotics (AMP-KF-FOX-P-AMC-CTT-SXT-DA-RD-QD-TEL-FD and AMP-KF-FOX-P-AMC-TE-CTT-DA-RD-QD-TEL-FD, respectively). In contrast, the most sensitive strain, 3763, showed resistance to only two antibiotics (FOX-TE). AMP-KF-FOX-P-AMC-RD was the most common antimicrobial resistant profile (91 of 294 strains). We also evaluated the multidrug resistance (MDR; Magiorakos et al., 2012) profiles and found that 95.6, 76.2, and 35.4% of isolates displayed simultaneous resistance to more than three, four, and five types of antimicrobials, respectively (Figure 2).

### Multilocus Sequence Typing and Cluster Analysis

**FIGURE 3:**
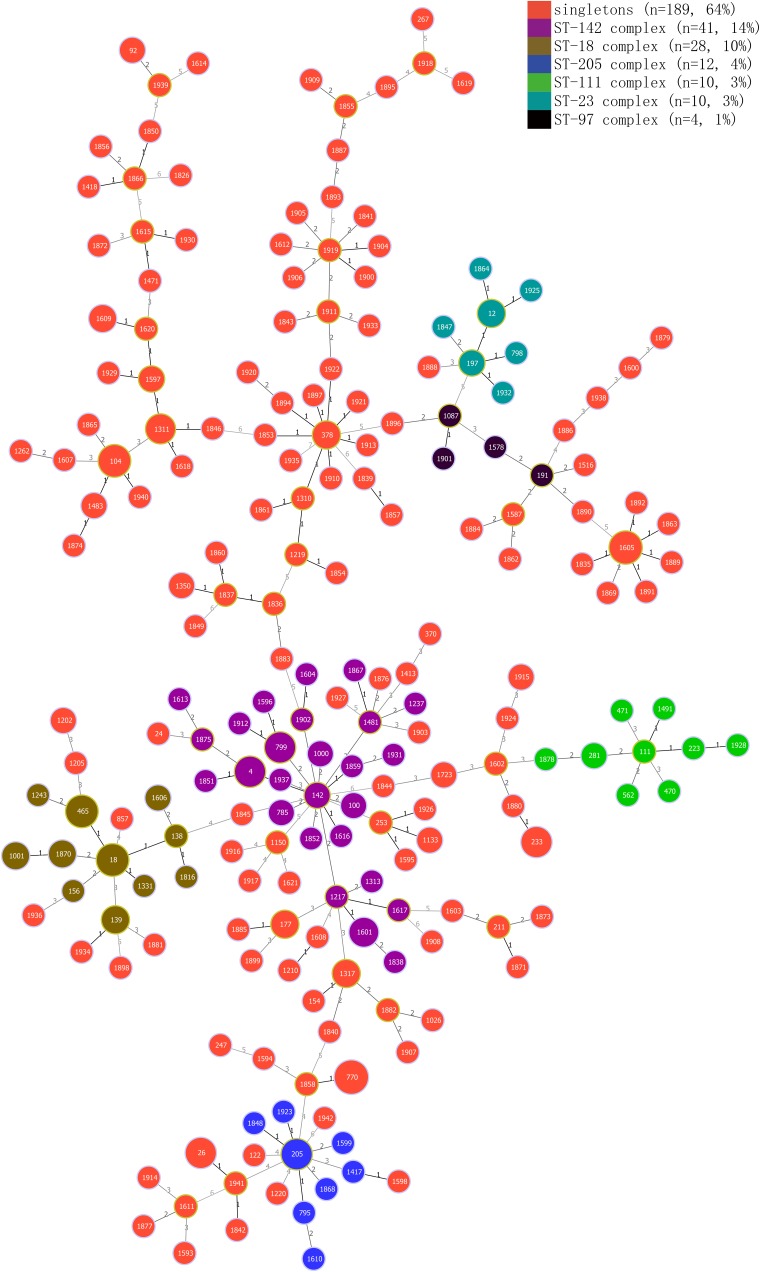
Minimum spanning tree and genetic diversity of *B. cereus* from vegetables. Different colors inside the circles represent different clonal complexes and singletons. The numbers inside the circles represent the corresponding sequence types (STs). Gradation of the line color and the corresponding number along the line represent variation of the seven loci between two strains at both ends of the line. The dominant STs are represented by the circles with larger diameters.

Genetic diversity was analyzed by the MLST method. Among all 294 strains, 210 STs were assigned, and 145 strains were assigned to 137 new STs. Additionally, 175 of all the 210 (83%) STs included a single strain, 35 STs included two to eight isolates, and only ST-770 included eight isolates, followed by ST-1605, which included seven strains. Five isolates belonged to ST-26, which is associated with clinical isolates. All 210 STs were grouped into six clonal complexes (CCs) and 189 singletons. The ST-142 complex was most frequent, including 41 isolates, while the ST-18, ST-23, ST-97, ST-111, and ST-205 complexes contained 28, 10, 4, 10, and 12 isolates, respectively (Figure 3).

## Discussion

### Prevalence of *B. cereus* Isolates in Vegetables

Few studies have evaluated pathogenic *B. cereus* in vegetables worldwide and no report has focused on the whole of China. In our study here, we found that 50% of all vegetable samples collected from 39 major cities in China contained *B. cereus*. The contamination level was more or less the same as those of previous surveys in other countries, i.e., 20–48% in Korea (Chon et al., 2015; Kim H. J. et al., 2016; Park et al., 2018), 57% in Mexico City (Flores-Urban et al., 2014), and 52% in the southeast of Spain (Valero et al., 2002). These reports, together with ours, indicate that *B. cereus* contamination in vegetables is common in several countries and suggest that consumption of vegetables contaminated with *B. cereus* is a potential health hazard (Valero et al., 2002; Kim Y. J. et al., 2016). The high level of contamination by *B. cereus* may be partly attributed to contact with soil or air during field planting (European Food Safety Authority [EFSA], 2005; Arnesen et al., 2008) and exposure to air during transportation and sale. Upon contamination, *B. cereus* may form a biofilm on the surface of vegetables, resulting in its persistent difficulty to be eliminated (Majed et al., 2016). *B. cereus*-positive samples may also contaminate other vegetables by contact transmission. According to the Microbiological Guidelines for Food of Hong Kong, China (Food and Environmental Hygiene Department, 2014), even though the amounts of *B. cereus* between 10^3^ and 10^5^ CFU/g in ready-to-eat foods are considered to be “acceptable,” they pose potential risks, and the raw materials, processing period, and environment should be examined to investigate the reason why these foods are contaminated; if the amounts of *B. cereus* in ready-to-eat foods are more than 10^5^ CFU/g, their quality is considered to be “unsatisfactory” and their sale should be stopped. The standards of microbiological limits for ready-to-eat foods in Australia and New Zealand (New South Wales Food Authority, 2009), however, stipulate that the “acceptable” level of *B. cereus* is 10^2^–10^3^ CFU/g, and an “unsatisfactory” level is 10^3^–10^4^ CFU/g. The United Kingdom microbiological testing standards (Health Protection Agency, 2009) for ready-to-eat foods stipulate that the “acceptable” level of *B. cereus* in ready-to-eat foods is 10^3^–10^5^ CFU/g, and a level of more than 10^5^ CFU/g of *B. cereus* is considered to be “unsatisfactory.” Of all the *B. cereus*-positive samples in this study, 9.95% (21/211), mainly of *C. sativum L* and var. *ramosa Hort.*, had contamination levels of more than 1,100 MPN/g. The contamination levels of these samples were at least at the “acceptable” level according to Hong Kong and United Kingdom standards and at the “unsatisfactory” level according to Australia and New Zealand standards. This suggests that *B. cereus*-contaminated vegetables pose a potential risk of causing foodborne disease, and care needs to be taken when consuming them directly or with minimal processing.

### Multilocus Sequence Typing and Genetic Diversity

MLST is a crucial epidemiological typing method based on the sequences of seven different housekeeping gene loci; it is used in studies of evolution and population diversity of *B. cereus* isolates (Erlendur et al., 2004; Cardazzo et al., 2008; Liu et al., 2017; Yang et al., 2017). In this study, we employed MLST to analyze genetic polymorphism in isolates from vegetables. Most of the isolates were assigned to singleton (Figure 3). The six CCs were distributed in separate samples, except ST-23 complex and ST-97 complex. ST-18 complex, ST-23 complex, ST-142 complex, and ST-205 complex even crossed with some singletons, indicating high genetic diversity of the isolates. However, we could not find any unique STs that existed in only one particular vegetable variety. Five strains, two of which were isolated from var. *ramosa Hort*, one from *C. sativum L*, and the remaining two from *C. sativus L.*, were assigned to ST26, the same molecular type of clinical isolates NC7401 and F4810/72 (Agata et al., 2002; Fricker et al., 2007). Three out of these five isolates were identified as potential emetic strains. As the preformed cereulide in foods is persistent and may also lead to food poisoning (Agata et al., 1995), there is a potential risk when consuming these vegetables directly or with minimal processing. Interestingly, we found that all isolates that belonged to the ST-18 complex, ST-97 complex, and ST-142 complex harbored the same virulence gene profile (*hblA*-*hblC*-*hblD*-*nheA*-*nheB*-*nheC*-*cytK*), while other CCs showed no such phenomenon. Additionally, 30 of 32 isolates belonging to ST-18 and ST-97 complexes showed resistance to cefotetan (CTT, 30 μg), whereas only 10 of 73 isolates belonging to the other four CCs showed similar resistance, which may be explained by the properties of founder clones of different CCs.

### Virulence Gene Detection and Potential Toxicity

Diarrhea caused by *B. cereus* is attributed to different enterotoxins produced by these strains in the small intestine (Jeßberger et al., 2015), including Hbl, Nhe (Lund and Granum, 1997), and CytK (Lund et al., 2000). In this study, we evaluated seven enterotoxin genes in *B. cereus*, and the positive rates of *nheABC*, *hblACD*, and *cytK* were 95, 81, and 87%, respectively (Figure 2 and Supplementary Table S1). The positive rates of *nheABC*, *hblACD*, and *cytK* were higher than those found in Korea (69.5, 41.7, and 74.2%; Park et al., 2018), but the *hblA* gene was detected in only 82% of isolates, which is lower than that reported in Mexico City (Flores-Urban et al., 2014). When considering different kinds of food, the *nheABC* frequency in our vegetables was lower than in Sunsik from Korea (Chon et al., 2012) or in pasteurized milk from China (Gao et al., 2018), but the *cytK* detection rate was much higher than those in rice and cereals from Korea (55%; Park et al., 2009), in Sunsik from Korea (77%; Chon et al., 2012), and in pasteurized milk from China (73%; Gao et al., 2018). Owing to the properties of foods consumed raw or processed minimally, the wider distribution of diarrheal *B. cereus* in these vegetables and their potential hazard cannot be neglected.

Emetic symptoms are caused by the emetic toxin cereulide. The positive rate of *cesB* was 3%, which is higher than those reported in Korea and Mexico City (Flores-Urban et al., 2014; Chon et al., 2015; Park et al., 2018), almost the same as that (2.9%) in Sunsik of Korea (Chon et al., 2012), but slightly lower than the 5% in pasteurized milk of China (Gao et al., 2018). Although the positive rate of *cesB* was quite low when compared with the rates of enterotoxins, cereulide is very persistent and heat-stable. Even emetic toxin remaining in sterilized food can cause emetic symptoms accordingly (Agata et al., 1995), the emetic isolates in these raw consuming vegetables are still potential risks.

### Antimicrobial Resistance of *B. cereus* Isolates

*B. cereus* infection may lead to diarrhea, vomiting, and even death (Hilliard et al., 2003; Evreux et al., 2007; Jean-Winoc et al., 2013; Ramarao et al., 2014). Antibiotic resistance test may provide a theoretical reference for the clinical treatment of *B. cereus* food poisoning and infection. Detailed antimicrobial resistance information of the isolates from vegetables is shown in Supplementary Table S2. More than 72.5% of all isolates were susceptible to six classes of antimicrobial agents, including aminoglycosides (CN, K), ketolide (TEL), glycopeptides (TEC), quinolones (CIP), phenylpropanol (C), tetracyclines (TE), and folate pathway inhibitors (SXT). A total of 60.2 and 99.7% of the strains were also susceptible to third-generation cephalosporin (CTT) and penems (IPM), respectively. More than 83.0% of the isolates showed resistance to other β-lactam antibiotics, including penicillins (AMP, P), β-Lactam/β-lactamase inhibitor combinations (AMC), cephems (KF, FOX), and ansamycins (RD). The high resistance of *B. cereus* to β-lactam antimicrobials has been widely reported and may be due to the synthesis of β-lactamase (Philippon et al., 1998; Chen et al., 2004; Park et al., 2018). Notably, 83.0% of the isolates showed resistance to rifampin (RD), which is much higher than the resistance rates of vegetables in Korea (only 48.7%; Park et al., 2018) and even higher than those of different kinds of foods, including rice and cereal (62%; Park et al., 2009), ready-to-eat foods (0%; Agwa et al., 2012), and traditional dairy products (0%; Owusu-Kwarteng et al., 2017). This may be ascribed to the usage of antibiotics in different countries or the evolution of strains toward rifampicin resistance. These results emphasize the need for caution when using β-lactams and ansamycins (such as RD) for the clinical treatment of *B. cereus*. On the other hand, 95.6, 76.2, and 35.4% of isolates showed resistance to more than three, four, and five classes of antibiotics simultaneously, respectively, suggesting the need to monitor multiple drug resistance in *B. cereus*.

## Conclusion

Vegetables such as *L. esculentum Mill.*, *C. sativus L.*, var. *ramosa Hort.*, and so on are usually consumed directly or with minimal processing, so potential hazards associated with *B. cereus*-contaminated vegetables should not be ignored. The results in this study revealed a high incidence of *B. cereus* in vegetable samples collected from across China, for the first time as we know. Of all the samples, 21/211 (9.95%), mainly of *C. sativum L* and var. *ramosa Hort.*, had contamination levels of more than 1,100 MPN/g. According to the Microbiological Guidelines for Food of Hong Kong, China (Food and Environmental Hygiene Department, 2014), the United Kingdom microbiological testing standards for ready-to-eat foods (Health Protection Agency, 2009), and the standards of microbiological limits for ready-to-eat foods in Australia and New Zealand (New South Wales Food Authority, 2009), the contamination levels of these samples were at the “acceptable” level according to Hong Kong and United Kingdom standards and at the “unsatisfactory” level according to the Australia and New Zealand standards. These contamination levels indicate a potential risk caused by the consumption of *B. cereus-*contaminated vegetables either directly or with minimal processing and should be kept in mind while assessing the quality of vegetables. If necessary, the reason for the contamination should also be traced. The pathogenic ST ST26 was detected in vegetable isolates. Seven enterotoxin genes associated with diarrheal symptoms were widespread among isolates, and the emetic toxin gene was also detected. In addition, most isolates were resistant to β-lactam antimicrobials, such as amoxicillin-clavulanic acid, penicillin, ampicillin, cephalothin, cefoxitin, and rifampin. Our results indicate a potential risk of consuming vegetables without sufficient processing and the increasing difficulties in eliminating *B. cereus* with antibiotics. To ensure the health and safety for the public, it is therefore necessary to develop new methods to prevent contamination and consequent potential foodborne outbreak induced by *B. cereus* in raw consuming vegetables.

## Author Contributions

QW, YD, JW, JMZ, and PY conceived the project and designed the experiments. PY, SY, HG, YZ, XL, JHZ, SW, QG, LX, HZ, RP, and TL performed the experiments. QW and YD supervised the project. PY and YD analyzed the data and wrote the manuscript. QW, JW, and YD complemented the writing.

## Conflict of Interest Statement

The authors declare that the research was conducted in the absence of any commercial or financial relationships that could be construed as a potential conflict of interest.

## Footnotes

1 http://pubmlst.org/bcereus/info/primers.shtml

## Supplementary Material

FIGURE S1 Sampling cities where the vegetables were collected.

TABLE S1 Prevalence of virulence genes in *B. cereus* isolated from vegetables in China.

TABLE S2 Results of antimicrobial resistance test for *B. cereus* isolates in the study.
