# Corrigendum: *Bacillus cereus* Isolated From Vegetables in China: Incidence, Genetic Diversity, Virulence Genes, and Antimicrobial Resistance

**DOI:** 10.3389/fmicb.2020.00848

**Published:** 2020-04-29

**Authors:** Pengfei Yu, Shubo Yu, Juan Wang, Hui Guo, Ying Zhang, Xiyu Liao, Junhui Zhang, Shi Wu, Qihui Gu, Liang Xue, Haiyan Zeng, Rui Pang, Tao Lei, Jumei Zhang, Qingping Wu, Yu Ding

**Affiliations:** ^1^Department of Food Science and Technology, Institute of Food Safety and Nutrition, Jinan University, Guangzhou, China; ^2^State Key Laboratory of Applied Microbiology Southern China, Guangdong Provincial Key Laboratory of Microbial Culture Collection and Application, Guangdong Open Laboratory of Applied Microbiology, Guangdong Institute of Microbiology, Guangzhou, China; ^3^College of Food Science, South China Agricultural University, Guangzhou, China

**Keywords:** *Bacillus cereus*, food-borne pathogen, vegetables, incidence, MLST

In the original article, the reference for Andreja et al., 2010 was incorrectly written as “Andreja, R., Mieke, U., Tine, C., Mark, H., and Johan, D. (2010). Prevalence and characterisation of *Bacillus cereus* in vacuum packed potato puree. *Int. J. Food Sci. Technol*. 41, 878–884. doi: 10.1111/j.1365-2621.2005.01129.x” It should be “Rajkovic, A., Uyttendaele, M., Courtens, T., Heyndrickx, M., and Debevere, J. (2006). Prevalence and characterisation of *Bacillus cereus* in vacuum packed potato puree. *Int. J. Food Sci. Technol*. 41, 878–884. doi: 10.1111/j.1365-2621.2005.01129.x” And the citation Andreja et al., 2010 in the INTRODUCTION Paragraph 1 should read: “(Rajkovic et al., 2006).”

The reference for Altayar and Sutherland, 2010 was incorrectly written as “Altayar, M., and Sutherland, A. D. (2010). *Bacillus cereus* is common in the environment but emetic toxin producing isolates are rare. *J. Appl. Microbiol*. 100, 7–14. doi: 10.1111/j.1365-2672.2005.02764.x” It should be “Altayar, M., and Sutherland, A. D. (2006). *Bacillus cereus* is common in the environment but emetic toxin producing isolates are rare. *J. Appl. Microbiol*. 100, 7–14. doi: 10.1111/j.1365-2672.2005.02764.x” And the citation Altayar and Sutherland, 2010 in the INTRODUCTION Paragraph 1 should read: “(Altayar and Sutherland, 2006).”

The reference for Wang and Zhang, 2013 was incorrectly written as “Wang, S., and Zhang, D.W. (2013). Analysis of bacterial foodborne disease outbreaks in China between 1994 and 2005. *FEMS Immunol. Med. Microbiol*. 51, 8–13. doi: 10.1111/j.1574-695x.2007.00305.x” It should be “Wang, S., Duan, H., Zhang, W., and Li, J. W. (2007). Analysis of bacterial foodborne disease outbreaks in China between 1994 and 2005. *FEMS Immunol. Med. Microbiol*. 51, 8–13. doi: 10.1111/j.1574-695X.2007.00305.x” And the citation Wang and Zhang, 2013 in the INTRODUCTION Paragraph 1 should read: “(Wang et al., 2007).”

The reference for Barbara et al., 2008 was incorrectly written as “Barbara, C., Enrico, N., Lisa, C., Leonardo, A., Tomaso, P., and Valerio, G. (2008). Multiple-locus sequence typing and analysis of toxin genes in *Bacillus cereus* food-borne isolates. *Appl. Environ. Microbiol*. 74, 850–860. doi: 10.1128/AEM.01495-07” It should be “Cardazzo, B., Negrisolo, E., Carraro, L., Alberghini, L., Patarnello, T., and Giaccone, V. (2008). Multiple-locus sequence typing and analysis of toxin genes in *Bacillus cereus* food-borne isolates. *Appl. Environ. Microbiol*. 74, 850–860. doi: 10.1128/aem.01495-07” And the citation Barbara et al., 2008 in the DISCUSSION Multilocus Sequence Typing and Genetic Diversity Paragraph 1 should be “(Cardazzo et al., 2008).”

In the original article **“**(Osimani et al., 2018)” and “(Fricker et al., 2007)**”** were not cited in the Introduction part (Paragraph 1).

In the original article, there was an error in the Introduction part (Paragraph 1). The strains in the literatures we referenced to were not all isolated from food poisoning outbreaks, so our statement “Outbreaks with vomiting and diarrheal syndromes caused by *B. cereus*” in the manuscript may lead to misunderstanding. Besides, we think that the references on the foodborne outbreaks and pathogenic concentration of *Bacillus cereus* were not specific enough, so we prefer to add two more references (Fricker et al., 2007; Osimani et al., 2018) for the first two sentences.

The corrections have been made to INTRODUCTION Paragraph 1:

“*Bacillus cereus* is a Gram-positive, spore-forming opportunistic pathogen that is widespread in different environments and known to cause foodborne outbreaks in humans (Bottone, 2010; Osimani et al., 2018). *B. cereus* in food products at concentrations exceeding 10^4^ spores or vegetative cells per gram can cause food poisoning (Ehling-Schulz et al., 2006; Fricker et al., 2007; Meldrum et al., 2009). Prevalence of potential emetic and diarrheal *B. cereus* in different foods has been reported in Finland (Shaheen et al., 2010), Belgium (Rajkovic et al., 2006), Thailand (Chitov et al., 2008), the United Kingdom (Altayar and Sutherland, 2006; Meldrum et al., 2009), the United States (Ankolekar et al., 2009), South Korea (Park et al., 2009), and Africa (Ouoba et al., 2008). *B. cereus* is also one of the most prevalent foodborne pathogens in France and China (Glasset et al., 2016; Paudyal et al., 2018). From 1994 to 2005, 1,082 food poisoning cases caused by foodborne pathogens had been reported in China. *B. cereus* caused 145 (13.4%) of these cases, leading to six deaths (Wang et al., 2007).

The authors apologize for these errors and state that this does not change the scientific conclusions of the article in any way. The original article has been updated.

## References

[B1] Altayar M. Sutherland A. D. (2006). *Bacillus cereus* is common in the environment but emetic toxin producing isolates are rare. J. Appl. Microbiol. 100, 7–14. 10.1111/j.1365-2672.2005.02764.x 16405680

[B2] Ankolekar C. Rahmati T. Labbé R. G. (2009). Detection of toxigenic *Bacillus cereus* and *Bacillus thuringiensis* spores in U.S. rice. Int. J. Food Microbiol. 128, 460–466. 10.1016/j.ijfoodmicro.2008.10.006 19027973

[B3] Bottone E. J. (2010). *Bacillus cereus*, a volatile human pathogen. Clin. Microbiol. Rev. 23, 382–398. 10.1128/CMR.00073-09 20375358 PMC2863360

[B4] Cardazzo B. Negrisolo E. Carraro L. Alberghini L. Patarnello T. Giaccone V. (2008). Multiple-locus sequence typing and analysis of toxin genes in *Bacillus cereus* food-borne isolates. Appl. Environ. Microbiol. 74, 850–860. 10.1128/aem.01495-07 18083872 PMC2227710

[B5] Chitov T. Dispan R. Kasinrerk W. (2008). Incidence and diarrhegenic potential of *Bacillus cereus* in pasteurized milk and cereal products in Thailand. J. Food Saf. 28, 467–481. 10.1111/j.1745-4565.2008.00125.x

[B6] Ehling-Schulz M. Guinebretiere M. H. Monthan A. Berge O. Fricker M. Svensson B. (2006). Toxin gene profiling of enterotoxic and emetic *Bacillus cereus*. FEMS Microbiol. Lett. 260, 232–240. 10.1111/j.1574-6968.2006.00320.x 16842349

[B7] Fricker M. Messelhäußer U. Busch U. Scherer S. Ehling-Schulz M. (2007). Diagnostic real-time PCR assays for the detection of emetic *Bacillus cereus* strains in foods and recent food-borne outbreaks. Appl. Environ. Microbiol. 73, 1892–1898. 10.1128/AEM.02219-06 17259359 PMC1828801

[B8] Glasset B. Herbin S. Guillier L. Cadelsix S. Vignaud M. Grout J.. (2016). *Bacillus cereus*-induced food-borne outbreaks in France, 2007 to 2014: epidemiology and genetic characterisation. Euro Surveill. 21:30413. 10.2807/1560-7917.ES.2016.21.48.30413 27934583 PMC5388111

[B9] Meldrum R. J. Little C. L. Sagoo S. Mithani V. Mclauchlin J. de Pinna E. (2009). Assessment of the microbiological safety of salad vegetables and sauces from kebab take-away restaurants in the United Kingdom. Food Microbiol. 26, 573–577. 10.1016/j.fm.2009.03.013 19527831

[B10] Osimani A. Aquilanti L. Clementi F. (2018). *Bacillus cereus* foodborne outbreaks in mass catering. Int. J. Hosp. Manag. 72, 145–153. 10.1016/j.ijhm.2018.01.013

[B11] Ouoba L. I. Thorsen L. Varnam A. H. (2008). Enterotoxins and emetic toxins production by *Bacillus cereus* and other species of *Bacillus* isolated from Soumbala and Bikalga, African alkaline fermented food condiments. Int. J. Food Microbiol. 124, 224–230. 10.1016/j.ijfoodmicro.2008.03.026 18474404

[B12] Park Y. B. Kim J. B. Shin S. W. Kim J. C. Cho S. H. Lee B. K.. (2009). Prevalence, genetic diversity, and antibiotic susceptibility of *Bacillus cereus* strains isolated from rice and cereals collected in Korea. J. Food Prot. 72, 612–617. 10.1089/cmb.2008.0063 19343952

[B13] Paudyal N. Pan H. Liao X. Zhang X. Li X. Fang W.. (2018). A meta-analysis of major foodborne pathogens in Chinese food commodities between 2006 and 2016. Foodborne Pathog. Dis. 15, 187–197. 10.1089/fpd.2017.2417 29652195

[B14] Rajkovic A. Uyttendaele M. Courtens T. Heyndrickx M. Debevere J. (2006). Prevalence and characterisation of *Bacillus cereus* in vacuum packed potato puree. Int. J. Food Sci. Technol. 41, 878–884. 10.1111/j.1365-2621.2005.01129.x

[B15] Shaheen R. Svensson B. Andersson M. A. Christiansson A. Salkinoja-Salonen M. (2010). Persistence strategies of *Bacillus cereus* spores isolated from dairy silo tanks. Food Microbiol. 27, 347–355. 10.1016/j.fm.2009.11.004 20227599

[B16] Wang S. Duan H. Zhang W. Li J. W. (2007). Analysis of bacterial foodborne disease outbreaks in China between 1994 and 2005. FEMS Immunol. Med. Microbiol. 51, 8–13. 10.1111/j.1574-695X.2007.00305.x 17666075

