# Supplementary figures and images for: Bacillus cereus Isolated From Vegetables in China: Incidence, Genetic Diversity, Virulence Genes, and Antimicrobial Resistance
Source: Front Microbiol. 2019 May 15;10:948. doi: 10.3389/fmicb.2019.00948 (PMC6530634; doi:10.3389/fmicb.2019.00948)

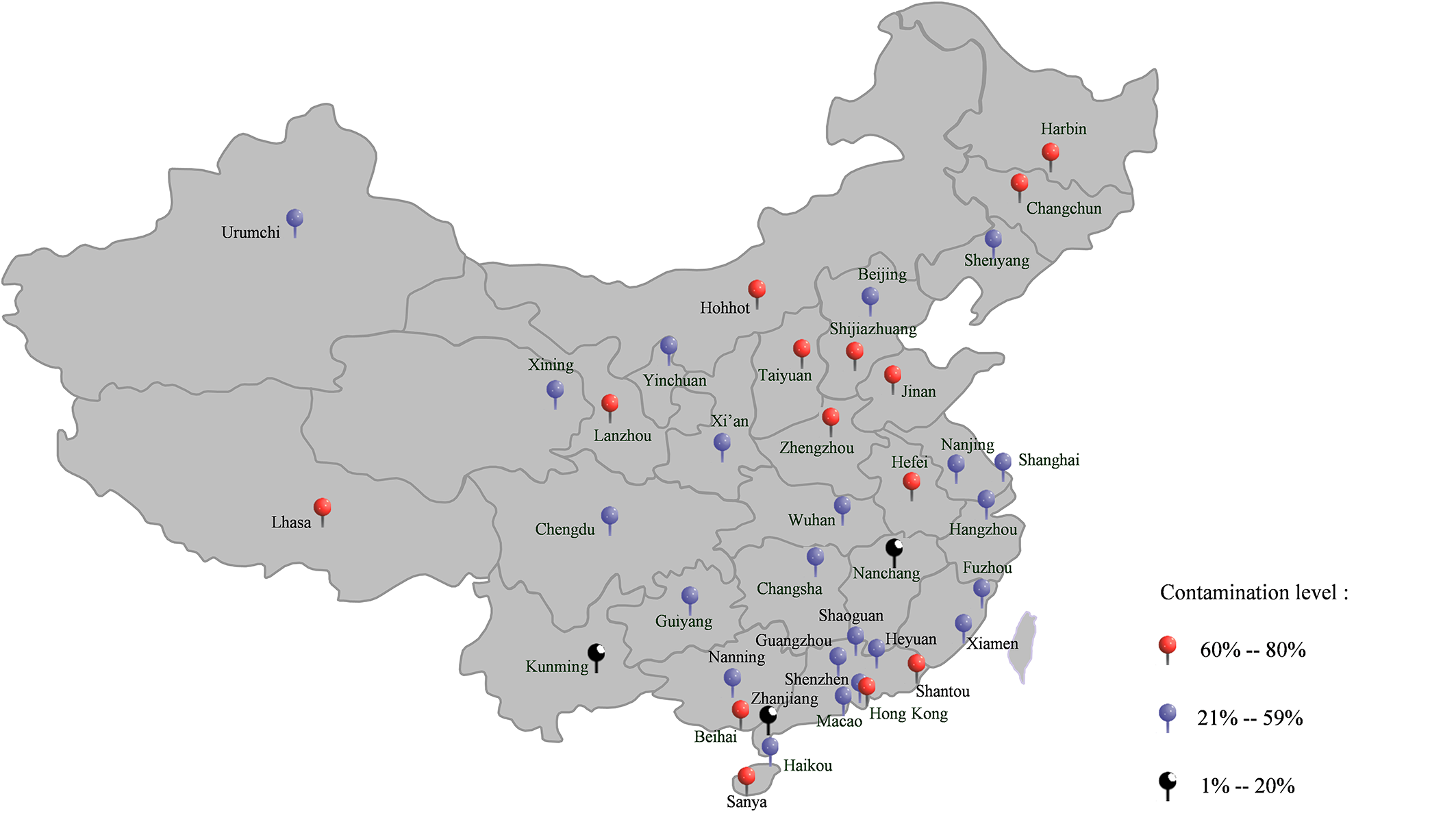

Supplement: FIGURE S1 — Sampling cities where the vegetables were collected. [file Image_1.TIF]
