# Supplementary material for: Bacillus cereus Isolated From Vegetables in China: Incidence, Genetic Diversity, Virulence Genes, and Antimicrobial Resistance
Source: Front Microbiol. 2019 May 15;10:948. doi: 10.3389/fmicb.2019.00948 (PMC6530634; doi:10.3389/fmicb.2019.00948)
Supplement: TABLE S1 — Prevalence of virulence genes in B. cereus isolated from vegetables in China. [file Table_1.doc]

**Supplementary Table 1 Prevalence of virulence genes in *B. cereus* isolated from vegetables in China.**

| **Toxigenic genes** | **Number of strains (%) positive for target gene** |
| --- | --- |
| Hemolysin BL genes |  |
| *hblA* | 241 (82%) |
| *hblC* | 264 (90%) |
| *hblD* | 265 (90%) |
| *hblACD* | 239 (81%) |
| Non-hemolytic enterotoxin genes |  |
| *nheA* | 292 (99%) |
| *nheB* | 293 (100%) |
| *nheC* | 282 (96%) |
| *nheABC* | 280 (95%) |
| Cytotoxin K gene |  |
| *cytK* | 256 (87%) |
| Cereulide synthetase gene |  |
| *cesB* | 8 (3%) |
| All eight toxin genes | 5 (2%) |
