# Supplementary material for: Bacillus cereus Isolated From Vegetables in China: Incidence, Genetic Diversity, Virulence Genes, and Antimicrobial Resistance
Source: Front Microbiol. 2019 May 15;10:948. doi: 10.3389/fmicb.2019.00948 (PMC6530634; doi:10.3389/fmicb.2019.00948)
Supplement: TABLE S2 — Results of antimicrobial resistance test for B. cereus isolates in the study. [file Table_2.DOC]

**Supplementary Table 2 Results of antimicrobial resistance test for *B. cereus* isolates in the study.**

| Category | Antimicrobial Class | Antimicrobials | *Bacillus cereus* (n = 294) | | |
| --- | --- | --- | --- | --- | --- |
|  |  |  | Resistant | Intermediate | Sensitive |
| β-Lactams | Ⅰ | Penicillins |  |  |  |
|  |  | Ampicillin (10 μg) | 293 (99.7%) | 0 (0.0%) | 1 (0.3%) |
|  |  | Penicillin (10 units) | 293 (99.7%) | 0 (0.0%) | 1 (0.3%) |
|  | Ⅱ | β-Lactam / β-lactamase inhibitor combinations |  |  |  |
|  |  | Amoxicillin-clavulanic acid (20 μg/10 μg) | 287 (97.6%) | 0 (0.0%) | 7 (2.4%) |
|  | Ⅲ | Cephems(parenteral) |  |  |  |
|  |  | Cephalothin (30 μg) | 255 (86.7%) | 24 (8.2%) | 15 (5.1%) |
|  |  | Cefoxitin (30 μg) | 281 (95.6%) | 0 (0.0%) | 13 (4.4%) |
|  |  | Cefotetan (30 μg) | 69 (23.5%) | 48 (16.3%) | 177 (60.2%) |
|  | Ⅳ | Penems |  |  |  |
|  |  | Imipenem (10 μg) | 1 (0.3%) | 0 (0.0%) | 293 (99.7%) |
| Non–β-Lactams | Ⅴ | Aminoglycosides |  |  |  |
|  |  | Gentamicin (10 μg) | 5 (1.7%) | 2 (0.7%) | 287 (97.6%) |
|  |  | Kanamycin (30 μg) | 8 (2.7%) | 41 (13.9%) | 245 (83.3%) |
|  | Ⅵ | Macrolides |  |  |  |
|  |  | Erythromycin (15 μg) | 11 (3.7%) | 100 (34.0%) | 183 (62.2%) |
|  | Ⅶ | Ketolide |  |  |  |
|  |  | Telithromycin (15 μg) | 19 (6.5%) | 26 (8.8%) | 249 (84.7%) |
|  | Ⅷ | Glycopeptides |  |  |  |
|  |  | Teicoplanin (30 μg) | 7 (2.4%) | 49 (16.7%) | 238 (81.0%) |
|  | Ⅸ | Quinolones |  |  |  |
|  |  | Ciprofloxacin (5 μg) | 2 (0.7%) | 19 (6.5%) | 273 (92.9%) |
|  | Ⅹ | Phenylpropanol |  |  |  |
|  |  | Chloramphenicol (30 μg) | 0 (0.0%) | 16 (5.4%) | 278 (94.6%) |
|  | Ⅺ | Tetracyclines |  |  |  |
|  |  | Tetracycline (30 μg) | 20 (6.8%) | 46 (15.6%) | 228 (77.6%) |
|  | Ⅻ | Folate pathway inhibitors |  |  |  |
|  |  | Trimethoprim-Sulfamethoxazole (1.25 μg/23.75 μg) | 69 (23.5%) | 12 (4.1%) | 213 (72.4%) |
|  | XIII | Lincosamides |  |  |  |
|  |  | Clindamycin (2 μg) | 22 (7.5%) | 220 (74.8%) | 52 (17.7%) |
|  | XIV | Ansamycins |  |  |  |
|  |  | Rifampin (5 μg) | 244 (83.0%) | 42 (14.3%) | 8 (2.7%) |
|  | XV | Streptogramins |  |  |  |
|  |  | Quinupristin (15 μg) | 38 (12.9%) | 182 (61.9%) | 74 (25.2%) |
|  | XVI | Nitrofurans |  |  |  |
|  |  | Nitrofurantoin (300 μg) | 19 (6.5%) | 93 (31.6%) | 182 (61.9%) |
| Multi-drug resistance |  | ≥ 3 Antimicrobia | 281 (95.6%) | - | - |
|  |  | ≥ 4 Antimicrobia | 224 (76.2%) | - | - |
|  |  | ≥ 5 Antimicrobia | 104 (35.4%) | - | - |
